# Textile Supplies for Hospitals

**Published:** 1916-04-29

**Authors:** 


### TEXTILE SUPPLIES FOR HOSPITALS.

### X. Sheets, Towels, and Table Linens.

The general characteristics of linen and the goods made from it have received incidental mention at several points in discussing the relative suitability of different fabrics for particular purposes. Briefly summarised, these characteristics may be set down as great length of fibre as compared with cotton, great strength but low elasticity, good conduction of heat indicated by the coolness of linen to the touch, a relatively high immunity from soilure in wear, and a comparatively poor affinity for dyestuffs. The length of the fibre and the strength under tensile strains are related closely to each other, the long-fibred linens being stronger than the short. The comparative coolness and cleanliness are at least in part traceable to the length and the arrangement of the fibres within the threads; the arrangement not being such as to contain warm air readily.

The linen fabrics in regular use include the plain linens known as "heavy" linens, from which aprons and surgical overalls are made. They have to be washed daily, and linen is preferred to cotton even at a materially higher price because it endures more washings, and a principal reason why linen bears laundering better lies in the fact that its fibres are many inches long, while those of cotton are only about one inch. The cheaper apron cloths are all-cotton, starched and finished to look like linen. The next best are union linen, in which a linen warp is substituted for a cotton one at some advance in price and with a real gain in strength. Apron cloths of pure linen are, however, to be preferred, and these rise in price according to their fineness. In the best grades the ultimate fibres are longer and better bound together in the thread, the threads themselves are finer and less irregular, and there are more threads to the square inch; so that the threads themselves as well as their component fibres are more securely held in the fabric.

### Bleached and Unbleached Linen.

Linens are either bleached or unbleached, and in the former case they may be bleached to different extents. Four grades of bleaching are recognised—"full," in which the goods are brought to a maximum of whiteness, "three-quarters," "half," and "quarter" bleached. In giving linen a full bleach there is a loss of about 20 per cent. in weight and of about 15 per cent. in half-bleaching. Whereas it is found that good bleaching materially strengthens cottons, the case is otherwise with linens. The explanation lies not so much in any difference of process as in the difference in the structure of the fibre. The unicellular cotton filaments are independent of each other first and last, but the multicellular flax fibre is bound together by vegetable gums which the process of bleaching removes. The loss of strength is one that can be afforded much better by linen than cotton, and it does not follow that the act of bleaching reduces the tensile strength of linens below any degree of strain to which they may be subjected in common use. In continuously washing and exposing unbleached linen to light a gradual process of bleaching occurs in course of wear, accompanied by a diminution of weight and of strength. In employing bleached linen the loss is incurred beforehand, and as bleached cloth is the more expensive, the loss tells in two directions. The logic of the facts is not difficult to read. At any rate, in buying the lower-priced, less closely twisted and woven linens, the unbleached is to be preferred, as in their case the consequent loss of strength tells with most effect. It is, however, apparent that the superior linens may be bleached without condemning them to a short life, and that, without bleaching, the full beauty of cloths intended for adornment as well as for utility is not revealed.

### Linen Towels.

The absorbency of linen is improved by bleaching, as the natural consequence of removing the protective layer of waxy matter from the underlying cellulose. To that extent, therefore, it is desirable to bleach linen towellings, but the consideration is offset by another. Towels are subjected to severe strains by a vigorous person drying his own hands, for example, and the retention of a maximum of strength in them is found worth some initial defects of appearance. The demands upon the strengths of towels vary with the purposes to which they are put, the greatest strength being required ordinarily from hand and roller towels. The standard specification in use by a large institution suggests the high strength that may advantageously be sought after:—

Specification for Huckaback Linen Towels, Unbleached.

Width.—25 inches.
Length.—3 feet 6 inches.
Weight per lineal yard.—8 to $8\frac{1}{4}$ oz.
Threads per inch warp.—64.
Threads per inch weft.—42.
Strength 9 in. × 4 in. warp.—650 lb.
9 in. between jaws weft.—400 lb.
Foreign matter.—Not exceeding 4 per cent.
Remarks.—Towels to be woven with $1\frac{3}{4}$-inch centre stripe of fast-dyed Turkey-red, showing the lettering in $1\frac{1}{2}$-inch letters.

If the strains of 650 and 400 pounds upon strips measuring 36 square inches appear to exceed any probable trials to be met in use, they are still below the average strength of towels made from "long line" flax in accordance with the given specification of weight and structure.

The name "huckaback" refers solely to the weave, and not at all to the material, denoting a structure which provides a firm groundwork and an irregular granular surface. The crossing of threads is arranged so that at intervals a thread

Note.—Previous articles in this series appeared on December 18, 1915, and January 8 and 22, February 5 and 19, March 4 and 18, and April 1 and 15, 1916. The next article will treat of "Surgical Dressings."

of weft floats above five threads of the warp, and these roughnesses on the surface facilitate the taking up of moisture and the reaction of the skin. Linen towels are also woven plain, as for glass-cloths, or twilled as in some roller towels. The twill is commonly made to expose upon the surface the weft threads, which as the less tightly twisted of the two are the more receptive of water. The Turkish towellings used after the bath are made both in linen and in cotton, and for pure absorbency, bleached cotton is upon the whole to be preferred. If cotton Turkish towels are bought it is more advisable to buy white ones than those which are dyed to simulate the brown colour of unbleached linen. The linen variety has the advantage in wear, as the pile does not disintegrate so quickly as a pile made with cotton, nor does the towel so soon break into holes. The pile of the Turkish towel is composed of loops, and these are formed in the loom by a special arrangement of the threads and mechanism In weaving ordinary cloth the whole warp is held under equal tension, but in making Turkish towels there are two warps, one tight and one slack. The weft is thrown across in the ordinary manner, but these threads are not beaten home as regularly as in ordinary weaving. Successive threads of weft are woven in open texture, and when eventually they are forced back, the taut warp forms the groundwork of the fabric and the slack warp is bent into loops. That which principally holds the cloth together is the short, or tight warp, and as it is desirable for this to be as strong as possible, a linen one is to be preferred.

### Table Linen.

The same main considerations apply to the table linens as to other linen goods. Where strength and cheapness are of more moment than elegance unbleached tablecloths and serviettes are chosen. The finest of damask linen is fine indeed, and a cloth of the very best quality may easily cost more in the first instance than all the silver and crockery that are placed upon it, and be a good investment at that. It is, however, the case that even the finest of linen damasks are liable to contain latent defects, imperceptible while the article is new and unwashed, but presently apparent in the form of cuts or holes. Linen, despite its strength, is relatively brittle, and in course of being finished for market it is calendered between heavy rollers. Should the fabric become crimped at any place the rollers are apt so to damage the cloth that a hole quickly appears in it for no apparent reason. Great pains are taken by linen finishers to avoid these mishaps, which nevertheless occur oftener than anybody wishes. The finest linen damask is a triumph of textile workmanship, made from very fine-spun yarns with a great many threads per inch and woven with large and ornate figures. These flowers or patterns are produced by a process of twilling, using what is known as the satin twill. To form the figures the warp threads are twilled on the surface, and in the groundwork the weft is brought to the face. The pattern exhibits itself as a play of light and shade, and in changing position before a damask-covered table it may be seen that what appeared as light when regarded at one angle shows as shade when seen from another. The solution is to be found in the direction of the twisting of the yarn. The warp threads are right-hand spirals and the weft left-hand, and they reflect light back to the eye correspondingly. Such is the system of damask weaving as carried out on Jacquard looms permitting of the separate control of many hundreds of threads. The principles are precisely the same in the coarser and unbleached cloths of the staff table, although some of these have a few bleached or mercerised threads introduced into them to enliven the pattern.

### The Identification of Linen.

The difference in price between linen and cotton makes the distinction between the two of some importance. Superficial observation and crude tests are somewhat inconclusive, and perhaps there is no ready means more reliable than an examination of the length of the fibre unravelled from yarn taken out of the fabric. The comparative strength is a good index, although some practice is required to judge of it. Under the microscope linen fibre is seen to be oval in section instead of round, and to contain joints and knots at intervals. Some conclusions can be drawn from tests made with the aid of dyestuffs, when the depth of the colour taken up distinguishes linen from cotton. When touched with a match linen burns with a fiercer flame than cotton, due to the presence within it of resinous matters. In one flame-experiment the fabric is first steeped in a concentrated solution of sugar and common salt. After the specimen has been dried, threads are dissected from the sample and are ignited separately, and whereas the cotton threads char to blackness, the linen ones carbonise to a grey ash. Again, a sample may be boiled in a weak solution of washing soda, dried, spread upon glass and saturated with oil. When held against the light the cotton threads appear opaque and the linen ones translucent. Attempts at distinguishing between all-linen and union linen have been made sometimes with the aid of drops of ink. The greater capillarity of the long linen fibre tends to produce an oval blot with its longer axis running in the direction in which the linen lies. The results obtained in practice, however, do not warrant implicit trust in the results observed.

The general merit of linen as a washing article is seen in its adoption for starched cuffs and collars and in its capacity as a washing fabric linen is at its best. The physical constitution which enables linen goods to withstand high breaking-strains when tested upon the machine serves linen in good stead in the laundry. The demand for articles stronger than the superficially similar ones made from cotton secures a special place for true linens in the economy of hospital management and in the household. The outward similarity leads to abuse. The sale of cotton goods as linen ones is contrary to the law, and severe measures have repeatedly been taken against offenders at the instance of Irish linen manufacturers.

